# State Lunatic Asylum, Austin

**Published:** 1892-12

**Authors:** 


STATE LUNATIC ASYLUM, AUSTIN.—Superintendent Dr. F. S. White has made his annual report to the Governor and Legislature; a copy is before us. It is handsomely illustrated with a cut of the splendid buildings—the superintendent's residence, views in the park, etc.—a new and commendable feature in such reports. The pamphlet is gotten out in a manner highly creditable, alike to the Superintendent and the State printers, B. C. Jones & Co.

Patients admitted during the year ending October 31, 96; discharged restored, 45; discharged improved, 29; discharged unimproved, 22; escaped, 2; died, 32; remaining on hand 334 males and 274 females,—total, 608. The per cent. of deaths is 4.04. Total amount expended during the year, $114,685.51; of this, $2,668.07 was for permanent repairs in consequence of the recent fire.

Amount received from private patients, $2,075.30; amount received for dairy products, $3,102.50; for farm products, $4,707.20, etc.

The general health of the asylum has been good. The Board of Managers have highly endorsed Dr. White's administration, and in their report speak in terms of the highest commendation of his ability and management.

